# The Use of Hebbian Cell Assemblies for Nonlinear Computation

**DOI:** 10.1038/srep12866

**Published:** 2015-08-07

**Authors:** Christian Tetzlaff, Sakyasingha Dasgupta, Tomas Kulvicius, Florentin Wörgötter

**Affiliations:** 1 Institute for Physics – Biophysics, Georg-August-University, Friedrich-Hund Platz 1, 37077, Göttingen, Germany; 2 Bernstein Center for Computational Neuroscience, Georg-August-University, Friedrich-Hund Platz 1, 37077, Göttingen, Germany

## Abstract

When learning a complex task our nervous system self-organizes large groups of neurons into coherent dynamic activity patterns. During this, a network with multiple, simultaneously active, and computationally powerful cell assemblies is created. How such ordered structures are formed while preserving a rich diversity of neural dynamics needed for computation is still unknown. Here we show that the combination of synaptic plasticity with the slower process of synaptic scaling achieves (i) the formation of cell assemblies and (ii) enhances the diversity of neural dynamics facilitating the learning of complex calculations. Due to synaptic scaling the dynamics of different cell assemblies do not interfere with each other. As a consequence, this type of self-organization allows executing a difficult, six degrees of freedom, manipulation task with a robot where assemblies need to learn computing complex non-linear transforms and – for execution – must cooperate with each other without interference. This mechanism, thus, permits the self-organization of computationally powerful sub-structures in dynamic networks for behavior control.

When we are performing a complex skill, like neatly stacking two blocks, our motor system needs to accurately control position and orientation of the hand, which took us quite some time to learn when we were children. During this process, we had learned different movements and how to combine them to more complex ones and we had formed memory representations for these movements, too. These representations are considered to be expressed by structure and activity of cell assemblies^1^, which are created by interactions of several plasticity mechanisms^2^,^3^,^4^ during learning. Several studies support this idea and suggest that the coordinated activity of cell assemblies results in motor fine-control^5^,^6^,^7^. During any motor task several thousands of neurons in many cell assemblies are active and perform complex non-linear calculations to control the different degrees of freedom of the involved limbs. Adults master a large number of motor skills requiring a multitude of different cell assemblies most – if not all – of which have been formed by learning. To achieve such mastery, our brain has to solve a very complex problem. It needs to create a large number of assemblies, which are computationally powerful, by using only a relatively small quantity of neurons for any of them. Furthermore, assemblies have to coexist without catastrophically interfering with each other. How this can be done based on unsupervised plasticity mechanisms is still widely unknown. Understanding this would, thus, carry substantial promise for our comprehension of how the brain can self-organize and provide the required requisite variety for complex motor control^8^.

It is known, on the one hand, that networks can be trained to perform complex non-linear calculations^9^,^10^,^11^, which could be used for motor control. This requires that those networks produce a reservoir of rich, transient dynamics from which the desired outputs can be siphoned off. This paradigm is known as Reservoir Network or Liquid State Machine Network computation^9^,^10^. For instance, proofs exist that a rich-enough dynamic network of this kind can emulate a Turing machine and, thus, provides general computational power^12^. Recent experimental evidence supports links between these complex transient dynamics and the dynamics of neuronal networks^13^,^14^,^15^.

On the other hand, based on the well-known synaptic-plasticity-and-memory hypothesis^16^,^17^,^18^, neurons can be linked together by strengthening their synapses depending on their neuronal activities, thereby, forming co-called Hebbian cell assemblies^1^. These cell assemblies are considered to be the basis of long-term memories^7^,^19^,^20^,^21^.

It appears, thus, straight-forward to combine these two concepts to arrive at the required computationally powerful cell assemblies. Alas, two effects can destroy such an approach. Self-organization of neurons into cell assemblies by the processes of synaptic plasticity induces ordered or even synchronized neuronal dynamics^1^,^6^,^22^,^23^. This will reduce the dynamics of the network often to a degree that the required requisite variety for complex calculations cannot be provided by it any longer^15^,^24^. In addition, trying to simultaneously create multiple assemblies will lead indeed to the aforementioned catastrophic interference if one cannot prevent them from growing into each other.

In this study, we exploit for the first time the interaction between neuronal and synaptic processes acting on different time scales to enable, on a long time scale, the self-organized formation of cell assemblies, while on a shorter time scale, to perform non-linear calculations. Note, our intention is to provide a rather general and mathematically sound mechanism that achieves stimulus driven self-organization of computational powerful entities (cell assemblies), but we do not attempt to model biological structures in any greater detail.

In this study, we first show how cell assemblies are formed by unsupervised plasticity processes and then we demonstrate that cell assembly growth will lead to improved computational power. For this, we use an example where a growing assembly is required to calculate some freely chosen non-linear transforms (e.g., power of seven of the input). Specifically we demonstrate that such a self-organized network is more efficient as compared to random networks. Furthermore, we show that under certain circumstances the outgrowth process guarantees that independently learned assemblies do not interfere with each other. This allows creating cooperative assemblies for the control of six degrees of freedom of an advanced robot for execution of a complex motor task.

## Results

**Figure 1:**
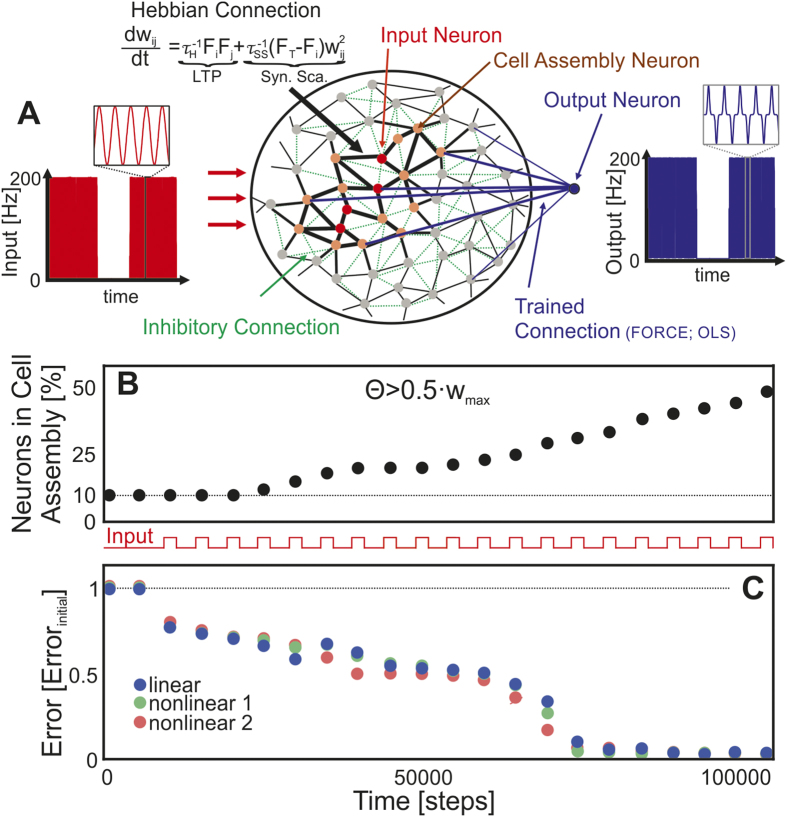
Cell assembly size and computational performance are correlated. (**A**) An input is delivered to several units (red disks) in a random neuronal network with very weak but plastic excitatory (black lines) and constant inhibitory connections (green dashed lines). The interaction of long-term potentiation (LTP) and synaptic scaling (Syn. Sca.) enables the formation of an Hebbian cell assembly (red and orange disks) by increasing synaptic efficacies (thicker lines) when repeating the input several times. Note this network is not topographically organized. The here shown neighborhood ordering is for graphical reasons only. Read-out units (blue disk) are connected (blue dashed) to the full network and trained in a conventional way to create the desired output (by FORCE^35^ or ordinary least squares (OLS)^9^). Here we used a single read-out unit, but several can be connected without additional constraints (see also Fig. 3). (**B**) With more learning trials the assembly grows and integrates more units. To measure this, we arbitrarily define assembly size by that set of units connected with efficacies larger than θ = 0.5·*W*_*max*_. (**C**) Parallel to the outgrowth of the cell assembly the error of the system to perform several linear and non-linear calculations decreases. linear: *O*(*t*) = *I*(*t*), *r* = −0.81; nonlinear 1: *O*(*t*) = *I*(*t*)^3^, *r* = −0.77; nonlinear 2: *O*(*t*) = *I*(*t*)^7^, *r* = −0.73; *O*(*t*): output; *I*(*t*): input; *r*: Pearson correlation coefficient between assembly size and error; please see Methods section for protocol details.

An external signal repeatedly stimulates several randomly chosen rate-coded input units in a randomly and very weakly excitatorily connected network with dominating inhibition (Fig. 1A and Methods section). The network units are defined in a way that they can serve as a model of single neurons or groups of neurons (see Methods). The goal is to set up such a system that repeated stimulation should – by synaptic plasticity – trigger (i) the formation and outgrowth of cell assemblies (group of strongly interconnected units) while (ii) still keeping them functionally separate. In other words, the excitatory synaptic efficacy *W*_*ij*_ between two units *i* and *j* has to be strong *within* an assembly and weak *between* them. These two requirements restrict the set of activity-dependent synaptic plasticity mechanisms (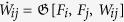 with pre- and post-synaptic activities *F*_*j*_ and *F*_*i*_ and weights *W*_*ij*_) as they have to fulfill the following fixed-point condition of the synaptic dynamics:


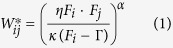


with offset Γ and parameters *κ*, *η*, and *α* (see Supplementary Text 1 for details on the derivation of this fixed point and parameter definitions).

To achieve this, a rule is needed where outgrowth by hebbian plasticity is compensated by a stabilization mechanism. Several such rules have been devised (Supplementary Text 1 and, e.g.,^3^,^25^,^26^,^27^), but there is an additional important constraint, that the known rules do not fulfill: It is also of central importance that outgrowth and assembly stabilization happen with appropriate timing. On the one hand, if stabilization is too fast it dampens synaptic development and leads to too small and computationally insignificant assemblies. On the other hand, if stabilization is too slow, synaptic assemblies are growing into each other as they are growing too large. In both cases the fixed point will be reached at non-optimal times.

Physiological data constrains the time scale of plasticity, which determines the outgrowth, and this is on the order of minutes to a few hours (LTP time scale^28^,^29^). Hence stabilization needs to be slower than that. For this reason here we are using synaptic scaling for stabilization, which acts on the scale of hours to days^30^,^31^,^32^ and – as shown next – arrives with nearly optimal duration at the above defined fixed point. Thus, for changing the connection weights in our network we define the following rule (see Methods and Supplementary Text 1):


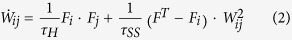


which combines (first term) hebbian synaptic plasticity^28^,^29^,^33^ with (second term) synaptic scaling^30^,^31^,^32^. Scaling acts stabilizing by trying to keep the system close to a desired firing rate *F*^*T*^ 30,34. Analytical results show (see Supplementary Text 1 for more details) that this rule leads to a system where stimulus-driven assemblies are formed by an outgrowth process such that a repeated stimulus will capture more and more units. Also the outgrowth speed can be estimated as:


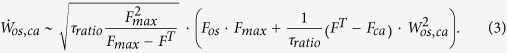


(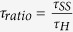: ratio of time scales of synaptic plasticity and scaling; *os*: outside assembly; *ca*: inside assembly; *F*_*max*_: maximum firing rate). From this equation the duration for reaching the fixed point can be derived as a function of the ratio of time scales of synaptic plasticity and scaling. Our model predicts that this curve has one minimum (Supplementary Text 1), where the fixed point is reached fastest, at a time-scale ratio of about 50–100, thus, if plasticity is about 50–100 times faster than scaling. Given the physiological time scales for synaptic plasticity, this leads to a prediction of a time scale of several hours for scaling, which is very much in line with the actually found slow characteristics of this process. Hence, the here chosen value for *τ*_*ss*_ of about 60·*τ*_*H*_ (time scale of hours^30^) seems a natural choice for the required stabilization mechanism, which leads to the speediest arrival at the required fixed point in such systems.

These results show that the here chosen rule can bring together two important and not necessarily compatible aspects for stable cell assembly formation, outgrowth and boundedness. Every stimulation induces weight strengthening and thereby the formation and outgrowth of a cell assembly starting from the input units (Fig. 1B). At the same time, due to the fixed point characteristics of this process, connection weights and – as a consequence – neural activities remain limited. Both are due to the interaction of hebbian synaptic plasticity with the slower mechanism of synaptic scaling^34^. This aspect will be of direct relevance when later on considering several assemblies in parallel. Importantly, due to the dominating inhibition, the here formed cell assembly does not serve as an attractor of the activity dynamics and it does not produce persistent activities^6^, which would be detrimental to computation^24^. Thus, with this type of repeated stimulation we obtain a growing assembly with transient activities (see also Supplementary Text 1).

Now we can analyse the computational power of such a structure during the growth process. To this end we interrupted assembly growth after each stimulus presentation and trained a read-out unit connected to the whole network to test the network’s ability to perform one of several linear or non-linear calculations^9^,^10^,^35^ and measured the error. Note, by using a linear read-out unit we ensure that the calculations are really only accomplished within the network. Interestingly, we observed that the performance of the network strongly correlates with the size of the cell assembly (compare Fig. 1B,C; average Pearson correlation coefficient from about 3000 data points: *r* = −0.77 ± 0.04; Supplementary Text 1, Fig. 1).

**Figure 2:**
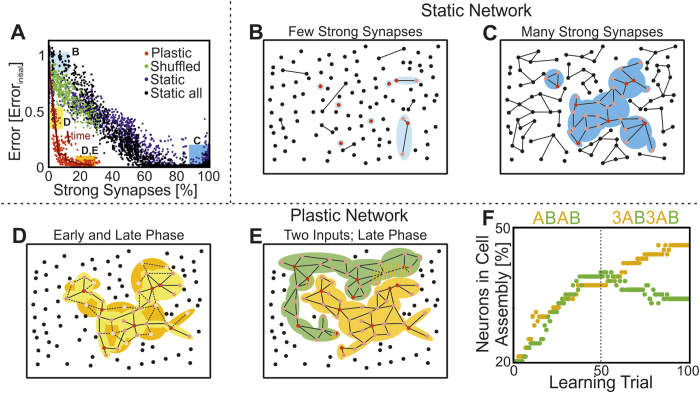
Comparison of the computational power of different networks relative to their synaptic structure. (**A**) The error in performing non-linear calculations (here nonlinear 1) decreases with the number of strong connection having a weight of *W* > 0.5·*W*_*max*_ in the network. We created 10 different network topologies and for each 300 randomly connected but unchanging networks (“static”) with 100 units each; plotting the error they produce against their number of strong connections (blue, black dots). Red dots show the error from networks that are obtained during the temporal development of assembly formation (temporal progress indicated by the arrow). The assembly network (red) needs far fewer strong connections as compared to the randomly connected static structures to achieve small errors (black: all units receive input; blue: same units as in the assembly network receive input). Shuffling the weights in the assembly network (green dots) leads to the same low performance as for the static networks demonstrating that random arrangements of the same strong connections does not suffice. Solid line indicates the development of one example of an adaptive network. (**B**–**E**) Schematic illustration of the underlying topology for different networks (red dots: stimulated units; orange dots: non-stimulated units driven by the input due to strong enough connections; black lines: strong connections; colored shadings: regions driven by the input). (**B**) static network with few strong connections, (**C**) static network with many strong connections, (**D**) plastic network after few learning trials (yellow shading) and after many learning trials (yellow and orange shading; dashed lines: strong connections obtained by longer learning). The color coded boxes in panel (**A**) show the errors for cases (**B**–**D**). (**E**) Schematic of a plastic network with two cell assemblies competing for units (striped areas). (**F**) Competitive development of the two competing cell assemblies “A” and “B” as a function of the input protocol (top).

Comparing the self-organized assembly network to networks with unchanging connections (“static” networks) shows that it is indeed the embedding of a strongly connected cell assembly that creates the computational power (see also Supplementary Text 1). Computational reservoirs are commonly generated using random connectivity of variable strength between many units^9^,^10^ and, as a result, weak and strong connections are randomly distributed therein. Inputs are also provided to randomly chosen units^9^,^10^. Consequentially, an input will lead to a spatially extended activity trace including many units if and only if one makes sure that there is a large-enough number of strong connections existing in the network to begin with. In these cases the error, when testing a computation, is indeed low. The data highlighted by a light blue box in Fig. 2A shows that randomly connected static networks with few strong connections (Fig. 2B) will perform poorly as compared to networks with many strong connections (dark blue box Fig. 2A and Fig. 2C), where the error is essentially zero. Remarkably, the same small error is obtained with an assembly network after some growth (Fig. 2D) with only a small fraction of strong connections, which can be seen when comparing the yellow (early learning) with the orange box (late learning) in Fig. 2A. The inputs provided to the network penetrate deep because strong connections exist mainly within the assembly which receives the input. Randomly shuffling the connections dissolves this effect (green dots) showing that really the creation of an assembly will lead to powerful computation. This is beneficial for several reasons. Limiting the number of randomly distributed strong connections mitigates the problem of persistent or synchronous activity^36^, which would entirely destroy computation^9^,^15^,^24^. In addition, possibly the most interesting property of such assemblies is that, due to the limited number of strong connections per assembly, several assemblies can coexist and/or compete in a realistic way with each other. As shown above and in previous studies^34^, synaptic scaling counterbalances the unrestricted growth processes of hebbian learning (LTP) guaranteeing that the system stays in the non-persistent activity regime^6^,^36^. But, in addition, scaling also implies competition between different presynaptic sites^37^,^38^. This leads to competition and/or coexistence between cell assemblies at the network scale. Hence, with an alternating but balanced presentation of two stimuli (“A”,“B”) two assemblies embedded in the same network grow in the same way (Fig. 2F before trial 50), but with dominant presentation of stimulus “A” the corresponding assembly will take over (Fig. 2F after trial 50) without interference between input traces. This is difficult to achieve in a random network as one has no control over the actual input trace configurations, which might easily randomly interfere and perturb each other^24^,^39^,^40^. Thus, the formation and outgrowth of cell assemblies by the interaction of synaptic plasticity and scaling enables an adequate, non-merging and self-organized allocation of computational resources (units and connections) to different inputs.

Motor control requires coordinated activation of many motor units. Evidence exists that this happens by subsequent triggering of muscle-synergies^41^,^42^, which control a subset of motor units into performing certain contraction patterns. Thus, multiple cell assemblies, embedded into the topography of the motor cortex, are involved in generating the correct activation sequence for the execution of a skill. Accordingly, when learning a new task, neural self-organization structures the network into the required assemblies. When practicing we check how far we have deviated from the desired goal and our nervous system derives from this an error signal used as feedback to guide the learning.

**Figure 3:**
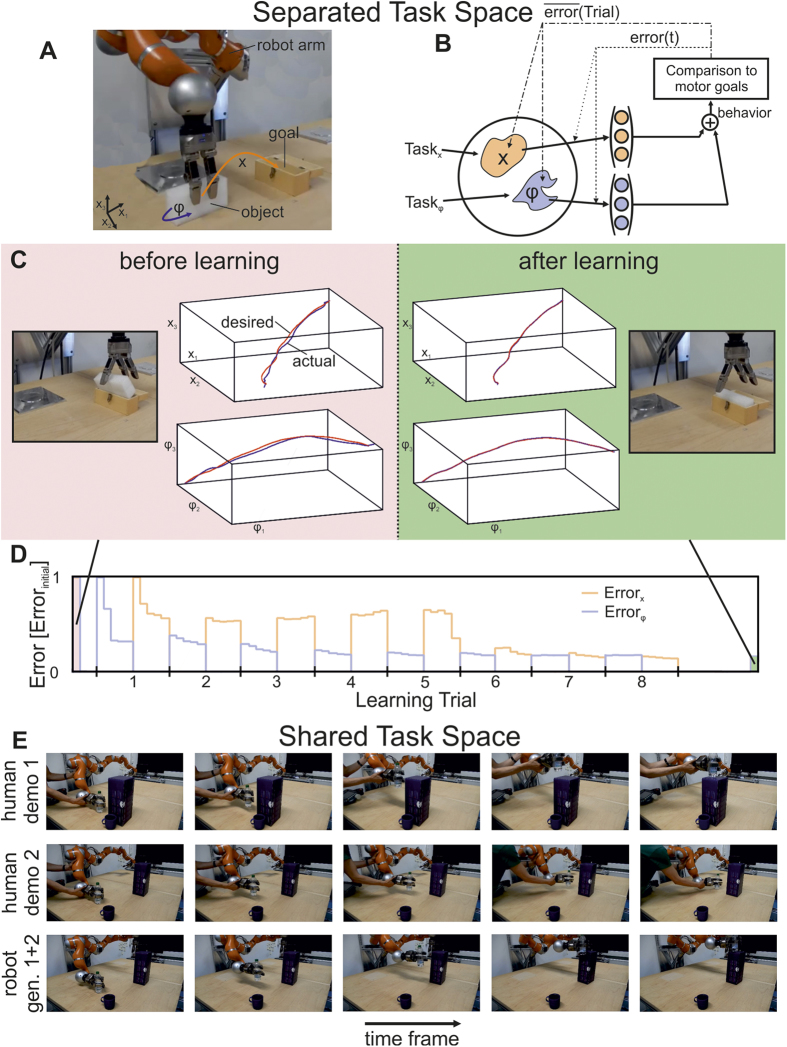
Highly accurate control of position and orientation in a robotic pick&place action is achieved by learning in coexistent, non-interfering assemblies. (**A**) For each trajectory for position task *x* and orientation task *ϕ*, three degrees of freedom need to be adjusted to an accuracy of about 1 mm on each side to make the white block fit into the brown box by the robot. A human has provided two example trajectories by guiding the robot arm; one for position-only and one for orientation-only, which are used for reference. Their trajectories are encoded by dynamic movement primitives (DMPs^44^,^45^, see Methods), which use Gaussian kernels for every DMP equally spaced along the trajectory. (**B**) Learning needs to adjust the amplitude of the kernels until success. For this we grow and train two assemblies using average and detailed trajectory error (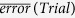, 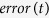), respectively. Learning of position (input: *Task*_*x*_) and orientation (*Task*_*ϕ*_) is done independently and alternatingly. (**C**) Robot performance and trajectories before and after learning. (**D**) Both errors (for *x* and *ϕ*) drop into the success range with only eight learning trials. (**E**) The network is also able to generalize movements with shared task space (the same 6 DoF of each single movement). Single movements are first demonstrated by a human (first and second row) and then the system combines both (last row). Please see Methods section for protocol details and Supplementary Videos.

A similar motor learning process can be shown in our network using a difficult task with a robot. Here we do not attempt to provide a detailed model of the human motor system. Rather we are concerned with the challenging problem to self-organize a network into functional assemblies under feedback error control. The error-signal determines the adaptation speed of the synaptic efficacies similar to three-factor learning rules (for a review see^43^). We were choosing an accurate pick&place problem, which is very difficult to learn for small children as well as machines. The task was to insert a block into a very tightly fitting box (Fig. 3A). To make this harder, we provide as reference signals for the learning only one single example action of putting the block into the box without having to rotate it and one other example of just rotating the block in right way and dropping it in the box without position change (Supplementary Videos 1–4). Creating a conjoint trajectory, thus, requires combining both these components. However, it can be observed that the robot fails by a substantial margin when it tries to do this prior to assembly growth (left panel in Fig. 3C). Will training assemblies lead to success? After all, it remains to be shown that there is no destructive cross interference occurring when several assemblies are active for generating a conjoint trajectory.

In order to solve this problem, we form and use two assemblies (Fig. 3B), one for the translation- and one for the rotation-movement and each computes one motor control function per degree of freedom (DoF) used for trajectory generation (in total 6 DoFs). Note, many more functions could be trained by using a larger network. This way a more direct match to the framework of muscle synergies could be enforced, which is not of relevance here, though.

Two learning modes are possible. It is possible to learn both task components (position and orientation) simultaneously (not shown), or one can let the network learn alternatingly to put or rotate the object without performing both components together. We are here showing the second variant, because it demonstrates more clearly how the system behaves and how coexistent assemblies are formed. Note, the here-used trajectory generation framework (dynamic movement primitives^44^,^45^) allows re-scaling and reshaping the learned trajectories for different configurations without having to re-learn from scratch. This is a long-known and important generalization property of such systems^45^ and not in the core of our discussions. Other trajectory control methods can be used to the same ends, for example also with Gaussian Mixture Models^46^.

Figure 3D shows that the error signal drives the outgrowth of the assemblies, which gets slower as the error decreases until the system reaches the minimal cell assembly sizes required to successfully complete the task. Thus, such adaptive networks, in contrast to static structures, generate an optimal trade-off between performance and resources used. Remarkably, these cell assemblies are formed to coexist without leading to interfering activities and the final, total error is similar to the error of the two independent components (green shaded box in Fig. 3D). This way, without learning this explicitly, a conjoint and accurate trajectory is being executed by the robot on co-activation of both assemblies after only 8 learning blocks (right panel in Fig. 3C).

Note, the joint spaces of both movements are in this case clearly seperated (rotation *ϕ or* translation *x*) and, therefore, each cell assembly can target different non-interfering read-out neurons. A possibly more interesting case is where learned actions happen in a shared joint space. For this the human demonstrated a mostly vertical placement movement of putting a bottle from the table on top of a box (Fig. 3E first row, Supplementary Video 5). In addition, he showed a left-right movement for replacing the bottle horizontally on the table (Fig. 3E second row, Supplementary Video 6). These two movements both use the same set of robotic joints. They are coupled in joint space. The question we asked was whether the system could learn to combine the two movements into a diagonal put-on-top motion. The network succeeds essentially in learning this after 8 learning blocks (Fig. 3E last row, Supplementary Video 7), but – when viewing the video – one sees that the resulting motion is not optimal. It starts a bit jerky and the box had been hit when trying to put down the bottle. Note the resulting motion had been obtained by directly combining the activity of both cell assemblies without further learning and this leads to not-fully complete generalization. This is very similar to humans trying to generalize a prior-learned action to a different situation. Commonly this leads to near-success and requires recalibration learning and/or feedback-loop correction (e.g. using vision). We did this with our system, too, and the final motion pattern after relearning is now accurate (Supplementary Video 8). Thus, these results show that the self-organized formation of non-merging powerful cell assemblies enables the independent learning of several complex tasks and, in addition, the parallel, non-interfering execution of them.

## Discussion

Previous works indicate that adaptation of synaptic efficacies influences computational performance in neural networks^24^,^35^,^39^,^40^,^47^, but the self-organization of a network into computationally powerful sub-structures poses a difficult and as yet unresolved problem.

In the current study efficient learning of a difficult motor skill was obtained by the combination of slow self-organization of the network into non-interfering cell assemblies with faster, error-driven acquisition of computational properties within these assemblies. As shown above, such systems are capable of combining substantial computational power with an economical use of network resources accommodating competition and/or coexistence of assemblies as determined by the inputs. In this study inputs are non-overlapping, thus, different inputs project to different disjunct groups of neurons. Overlapping inputs are beyond the scope of this study as they lead to very complicated neuronal and synaptic dynamics inducing possibly the destabilization of cell assemblies^6^. We expect that such disruptive effects could be eliminated by introducing plastic input synapses, which would sort assemblies according to their input features similar to (for example) self-organizing maps^3^,^48^,^49^,^50^. Such additional mechanisms could be used to reduce or eliminate any overlap. At that point of the investigation we conclude that the here presented dynamic network restructuring process is novel and not possible in static networks but that the problem of overlap will have to be the topic of future work.

Structure, viz. cell-assembly-, formation must be adaptive but not volatile, which suggests that synaptic plasticity needs to be stabilized by processes that act on longer time scales – such as synaptic scaling. Our analytical results show that the combination of plasticity and scaling is indeed especially well suited to achieve coexistent assemblies and that it is difficult to achieve this by other synaptic plasticity rules (see above and^6^,^34^). Other mechanisms as inhibition^3^,^51^ or short-term plasticity^3^,^52^ can also introduce local competition or partially stabilize neuronal dynamics, however, whether this holds also at network level and whether the resulting dynamics yields the formation of non-merging cell assemblies is still unknown. Specifically, it seems that plasticity + scaling also leads to the right temporal behavior where arrival at the fixed point happens with the expected physiological time scales (Supplementary Text 1).

In our study this had been used to create assemblies that can perform several non-linear computations and we had shown that difficult motor tasks can be encoded this way. To this end we had used a framework from robotics (DMPs) to encode trajectories by the time-series of learned DMP-kernel values. This carries a certain analogy to the sequential activation muscle synergies^53^ but falls short of any more detailed simulation of the brain’s motor system, which has not been intended by this work. We had used the method of kinesthetic guidance of the robot to obtain our reference signals for learning. Humans, on the other hand, mostly perform imitation learning instead. We had done this in a different context in some of our older works^54^, but for the current study this has been avoided as it only adds quite some complexity to the required generation of the reference signals, whereas it does not contribute to our core questions. The last robotic experiment (Fig. 3E) shows that even interfering signals can be combined into a conjoint movement, but that this combination remains suboptimal. This *looks* very similar to our own motor learning where generalization of learned movements for the creation of a new one is usually incomplete and erroneous. We employ sensory feedback (often vision) and more learning to arrive at better results and the same was needed for our artificial network to perform correctly.

It is known that synaptic plasticity and scaling act in many cortical areas^28^,^29^,^55^ and, thus, the interaction between cell assembly formation and their transient dynamics is not restricted to the here chosen examples. This is supported by the fact that in the model, the underlying topology has only a minor influence on the formation of cell assemblies and the emerging transient dynamics. Thus, we expect that the here presented effects may hold for wide variety of brain areas independent of their detailed topology. For instance, experiments and models provide evidence for the existence of transient dynamics^14^ within cell assemblies^56^ in the prefrontal cortex, too.

An important additional aspect of such systems is that they will stay in a non-persistent activity regime without which computations will deteriorate. Note that this constraint is equivalent to the condition that the spectral radius is smaller than one in echo-state networks^10^. The transient activity present in our system is probably equivalent to the asynchronous irregular (AI) state in spiking networks^36^, which, in the same way, provides rich and transient dynamics^57^. This state is dominated by inhibition and, therefore, does not need any fine-tuning between inhibition and excitation^47^; a strong property which is also shared by the here presented system. In particular, experimental data from delayed vibrotactile discrimination tasks are best described by a combined model of cell assemblies and transient dynamics^13^. Thus, cell assemblies with transient neural dynamics seems to be ubiquitous feature in neural systems and the here presented results allow a better understanding of how such structures might be dynamically shaped into computationally powerful assembly networks.

## Methods

### Neuron and network model

The neuronal circuit we are using in each simulation consists of *N* units randomly connected with probability *p*^*E*^ for excitatory and *p*^*I*^ for inhibitory connections. *p*^*E*^ and *p*^*I*^ define the excitatory (*C*^*E*^) and inhibitory (*C*^*I*^) topology of the network. Each unit *i* of the network is described by a leaky membrane potential *u*_*i*_ and a firing rate *F*_*i*_ which depends nonlinearly on the unit’s actual membrane potential, *F*_*i*_[*t*] = Φ(*u*_*i*_[*t*]). This formulation allows for a general interpretation of each unit as either a rate-coded neuron or a population of neurons^58^. Thus, the here presented results are independent of the spatial scale of the neural circuit.

Each network unit has a membrane potential *u*_*i*_ changing with time constant *τ*_*u*_ and resistance *R*:


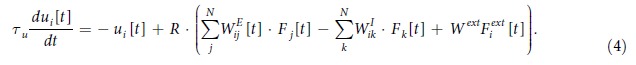


Each unit receives a weighted (*W*^*ext*^) noisy external input 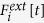 drawn from a Gaussian distribution with constant mean (*μ* = 0) and standard deviation (*σ* = 20) if not stated differently. The inhibitory inputs from the network are weighted by 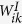 which are constant over time (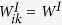 if 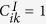 and 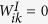 if 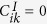). Excitatory connections 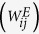 are plastic depending on the interaction of synaptic plasticity and scaling while keeping the underlying topology *C*^*E*^ (thus, 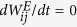 and 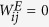 if 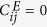). For the firing rate *F*_*i*_[*t*], without loss of generality, we use a sigmoidal transfer function


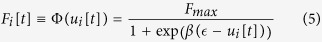


with maximal firing rate *F*_*max*_, steepness *β*, and inflection point ε**.

We simulated the network by the Euler method with step size Δ*t* = 0.3 in Matlab. Please find all parameters in Table 1.

### Synaptic plasticity and scaling

The excitatory connections of the network are plastic using an interaction of synaptic plasticity (here, long-term potentiation [LTP]^28^,^29^,^33^) and synaptic scaling^6^,^30^,^34^. Thus, the synaptic efficacy 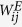 (also named *W*_*ij*_) between unit *i* and *j* is adapted in the following way:


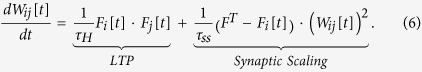


The LTP-term consists of the multiplication of pre- and postsynaptic activities *F*_*j*_ and *F*_*i*_ with time scale *τ*_*H*_. Synaptic scaling is a homeostatic process^59^ that drives the synaptic efficacies by the difference between actual *F*_*i*_ and the desired rate *F*^*T*^. As synaptic scaling is a slower process than plasticity (approx. hours to days^55^ as compared to minutes^28^), it depends on the time constant 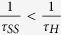. The weight dependency in the scaling-term (experimentally supported by^30^) guarantees that the synaptic weights are bounded between zero and 
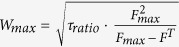
6,34 with 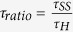 as the ratio of plasticity and scaling time constants.

### Stimulation protocols

For the formation of a cell assembly a group of units or neurons has to be stimulated together^6^. This stimulation induces, via the interaction of synaptic plasticity and scaling, changes of the corresponding synaptic efficacies. During non-stimulation all units receive a random input. First, we let the system relax according to this random input. Then, the external stimulation is given to the network (Fig. 1 and Supplementary Text 1). Each learning trial has a duration of 5000 time steps and consists of 2000 steps random input to all units and 3000 steps stimulation to ten units and random input to the others.

The exact temporal structure of the input-stimulation is irrelevant^6^. It only needs to be “variable enough” to drive the reservoir. Here, we use a simple sin-wave 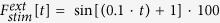 applied at every learning trial to the same subset of units (randomly chosen). To form a second assembly, another subset of units also receives a stimulation input. The learning trials are alternating (see Fig. 2).

### Cell assembly analysis

There are several ways possible to characterize a cell assembly^1^,^21^,^22^,^60^. In this work, we focus on the definition that a cell assembly is determined by the strength of the synaptic efficacies connecting groups of neurons^1^,^6^. Here, we define that, if the neurons or units are strongly connected with each other, they form a cell assembly. Thus, to find cell assemblies in the network, we define a threshold *θ* saying that synaptic efficacies larger than *θ* are ‘strong’ and others are ‘weak’ (w.l.o.g., *θ* = 0.5·*W*_*max*_). Importantly, the exact value of the threshold does not alter our results (Supplementary Text 1).

**Figure 4:**
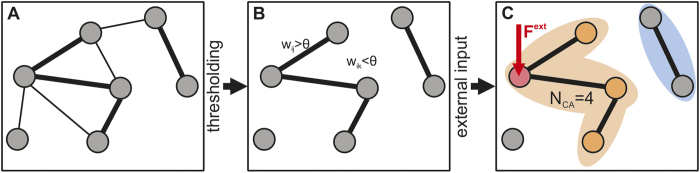
Analyzing cell assemblies. (**A**) Schematic illustration of a part of a network with connections of different efficacies (thickness of lines). (**B**) First we define a cell assembly as consisting of units connected by weights above threshold *θ*. Hence we delete all other connections. (**C**) Then, by starting at the units which receive external input (red), we count how many units can be reached in the thresholded network (orange). These units form an *input-dependent* cell assembly. Other groups of units with strong connections are classified as *other* assemblies (blue).

To determine the size of a cell assembly, we take the (excitatory) network (Fig. 4A) and delete all connections smaller than *θ* (Fig. 4B). The resulting network includes all potential cell assemblies for this particular *θ*-threshold. As we are interested in the input-dependent cell assemblies, in the next step, we determine those units receiving external stimulation (red in Fig. 4C). Starting from them, we follow the paths of strong connections (*w* > *θ*) and count how many units in the network can be reached along these paths including the stimulated ones (orange; in this example the size of the cell assembly is *N*_*CA*_ = 4). All other strong connections and their corresponding units (e.g., blue assembly in Fig. 4C) are counted as different cell assemblies (as, for instance, in Fig. 2E,F).

For calculating the correlation between the cell assembly size *N*_*CA*_ and the performance of the network to solve a given task (represented by the error *e*_*task*_), we calculated the Pearson correlation coefficient *r* defined as follows:


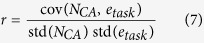


with cov(·, ·) as the covariance matrix and std(·) as standard deviation.

### Static networks

To classify the efficiency of the adaptive assembly network (Fig. 2A), we compared its performance to that of random networks with unchanging connections (“static networks”). For this, we created 10 different topologies and for each 300 different static networks with same inhibitory (*C*^*I*^) and excitatory (*C*^*E*^) topology. Only the excitatory synaptic efficacies are randomly distributed over the fixed topology. For each network, efficacies were drawn from a Gaussian distribution with a different mean 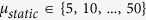 and standard deviation 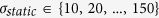 to get a diverse set of static networks. We truncated the distributions at *W* = 0 and *W* = *W*_*max*_.

### Read-out units

In this work we analyze the performance of a network when solving nonlinear tasks and how this changes with ongoing plasticity leading to cell assembly outgrowth. To achieve this, at different moments of network development, plasticity is stopped and the network’s performance is tested. After such a growth-stop, the network is driven by an external signal to the same units which have been stimulated during learning. Without loss of generality, this signal is the same as the learning signal. To assess the performance of the network each unit *i* is connected with strength 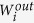 to a linear read-out unit. As a result, the activity of the read-out unit is given by


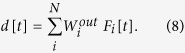


The resulting activity *d*[*t*] of the read-out unit is compared to the desired output signal 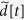 of the task. This determines the task-dependent error 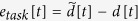. The error is used to adapt the connections between network and read-out unit in a supervised manner^9^,^10^,^35^. Here we use two different adaptation methods shown in the following: first-order reduced and controlled error (FORCE^35^) and ordinary least squares (OLS).

### FORCE

In the FORCE-algorithm^35^ the adaptation rate itself is adaptive according to the network activity. Consider in the following **f** as the vector with all activities of the network units as elements 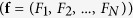 and **w**_**out**_ as vector of all output weights 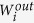. Then, the output weights are adapted as in the following:


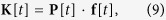



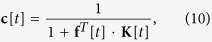



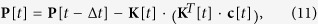



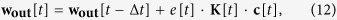


with 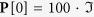 (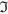 is the identity matrix). Here **P** is an NxN matrix proportional to the inverse of the correlation matrix of the network firing rate vector **f**. This update is done until the error converges (about 500 time steps). The absolute averages of the performance error *e*_*task*_[*t*] over the last 100 time steps are shown in Fig. 1C.

### OLS

In the motor task the error is feed back to the network controlling the speed of synaptic adaptation. This could result to dramatic changes in neural activities. As the FORCE-algorithm is very sensitive to such activity changes, here, the output weights, connecting the network units with the read-out units, are updated according to the ordinary least squares algorithm given in the following:


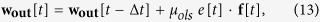


with update rate *μ*_*ols*_.

### Motor task

To solve the complex motor task of putting the block into the box (Fig. 3), we need to learn and control six degrees of freedom (DoFs). We use the well-established framework of dynamic movement primitives (DMPs;^44^) and define one DMP per DoF. Hence every DMP encodes the trajectory of one joint and complete movements are created as a linear composition of 6 DMPs. Note, there are many other possible ways to achieve the same and DMPs were chosen, because of their simplicity and because they are structurally quite close to the concept of muscle synergies^41^,^42^. The DMP formalism is provided below. For now, it suffices to know that a DMP receives its shape from a set of subsequently active kernels with different amplitudes, which creates the shape of the trajectory. Thus, the amplitudes of these kernels form a time series 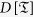, where 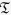 represents the location (moment of being active) of a given kernel on the trajectory.

Our goal is to form cell assemblies that can create such time series and then perform the motor task without error. To make this more difficult, we split the task into two subtasks, translation and rotation. We learn them *independently* wanting to show that their combination will nonetheless lead to success. Thus, the system forms two cell assemblies each encoding one of the subtasks (see Supplementary Text 1, Fig. 2, translation: *x*, orange; rotation: *ϕ*, blue). In Fig. 3E, both subtasks consists of rotation and translation movements thereby sharing the same task space. However, six time series *D*_*k*_ 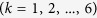 are extracted from the whole network by six read-out units.

Learning consists always of two processes: 1) assembly outgrowth by hebbian plasticity and synaptic scaling and 2) supervised adaptation (reservoir adaptation) of the output weight vectors 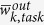 from the network to the read-out units in order to create the trajectory. Both processes are driven by error functions. To obtain those we need a reference signal. Hence, first one example of rotating the block into the slot (without translation) and one example of translating it (without rotation) was demonstrated by a human moving the robot arm manually into the correct position (kinesthetic guidance). Then the resulting time-series 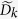 where extracted and represent the “desired values” to be reached by the learning (indicated by the tilde). Errors can then be calculated relative to these desired values.

We start by providing inputs to ten units each for a sub-task. They form the starting configuration of each assembly. The system is stimulated by two independent signals *Task*_*x*_ and *Task*_*ϕ*_, which allows driving both assemblies independently from each other. *Task*_*x*_ and *Task*_*ϕ*_ take the form of two different sin-waves (see Table 2), but it is only of relevance that these signals create a non-constant drive of the system and their actual shapes do not matter.

First we take the configuration as is – hence without any assembly growth – and calculate its performance by performing output weight adaption once. This is done by driving the assemblies with their task signals and calculating the inner-errors 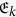 between desired and currently existing time-series (Supplementary Text 1, Fig. 2): 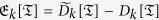. Note these errors are time-resolved along 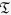 and, therefore, allow for the learning of a time-series as is common in reservoir networks. Hence, these errors are used to update the corresponding output weight vectors 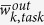 for the six output units *k* of both assemblies by the OLS algorithm. After this single update we tested the combined behavior of the robot, the result of which is found in Fig. 3C left, which shows that the machine did not succeed.

Next we let the assemblies grow. To control this we use the external error ε, which is the average (time-collapsed) error between desired and actual trajectories. To obtain this, time-series *D*_*k*_ are used to create the corresponding DMPs, which define the trajectories *T*. We calculate the temporal average Euclidean distance between *T*[*t*] and 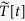 as (for translation): 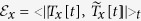. The final external error 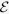 is given by the maximum of the translation and rotation errors 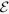
= max (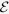
_*x*_, 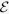
_*ϕ*_).

This is a behavior-dependent feedback signal and can be used to control assembly growth by using it as an additional factor in the learning rule Equation (6), which changes to:


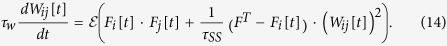


In general, this formulation is comparable to a third factor learning rule common, for example, in reinforcement learning^43^. Assembly outgrowth stops as soon as behavior is successful, hence when ε drops to zero.

After one outgrowth step we again perform output weight vector adaptation and measure the performance and so on. Ten of such learning trials are combined to one learning block. Note, the sequentiality of this procedure is owed to the use of computers. In a real network both processes (outgrowth and output weight vector adaptation) can possibly happen in parallel, but this is not of relevance for the purpose of this study.

What is of relevance though is that in general all *learning is performed alternatingly* between both assemblies, triggered by the respective *Task*_*x*/*ϕ*_ signals. This was done to show the intrinsic capability of such systems to balance assembly cooperation with competition.

### Dynamic movement primitives

To describe each 1-dimensional joint trajectory we use the well established framework of dynamic movement primitives (DMP^44^) as they assure fast convergence and generalization of movements^45^. Modified DMPs are based on a set of differential equations basically describing two dynamical systems^44^,^45^ the transformation and the canonical system.

The transformation system is formalized as follows:


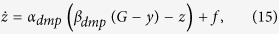



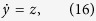



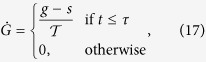


with *α*_*dmp*_ and *β*_*dmp*_ as time constants and 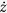, 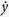 and *y* corresponding to acceleration, velocity and position, respectively. Here, *G* defines a goal function with *s* as start and *g* as goal states (start/end-point). 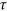 is the total duration of the movement. Initially we set *y*[0] = *G*[0] = *s*.

The canonical system is described by a sigmoidal-decay function:


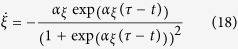


with time constant *α*_*ξ*_ defining the steepness of the function centered around 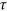. Initially we set *ξ*[0] = 1. The nonlinear function *f* is given by


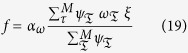


with scaling factor *α*_*ω*_ and


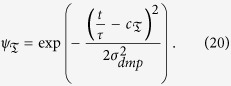



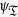
 are Gaussian kernels which are placed equidistantly along the trajectory in time and space with kernel centers 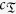 and width 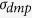. The shape of the movement is determined by the kernel weights 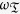. They are, on the one side, defined by human demonstration 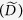 and, on the other side, learned by the adaptive network (*D*). Thus, the activity of the corresponding output unit *D*_*xd*/*ϕd*_ at time point 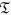 represents the weight of kernel 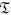.

## Additional Information

**How to cite this article**: Tetzlaff, C. *et al.* The Use of Hebbian Cell Assemblies for Nonlinear Computation. *Sci. Rep.* **5**, 12866; doi: 10.1038/srep12866 (2015).

## Supplementary Material

Supplementary Text 1

Supplementary Video 1

Supplementary Video 2

Supplementary Video 3

Supplementary Video 4

Supplementary Video 5

Supplementary Video 6

Supplementary Video 7

Supplementary Video 8

## Figures and Tables

**Table 1:** This table contains all parameters and their values for the network if not stated elsewhere differently.

Parameters		
Name	Abbreviation	Value
unit number	*N*	100
excitatory connection probability	*p*^*E*^	0.1
inhibitory connection probability	*p*^*I*^	0.2
inhibitory efficiency	*W*^*I*^	0.3·*W*_*max*_
external efficiency	*W*^*ext*^	*W*_*max*_
maximal firing rate	*F*_*max*_	100
steepness	*β*	0.03
reflection point	*ε*	120
resistance	*R*	0.012
membrane time constant	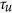	1
LTP time constant	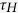	3·10^4^
time constant ratio	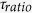	60
desired activity	*F*^*T*^	1

**Table 2:** This table contains all parameters and their values for the motor learning task.

Name	Abbreviation	Value
task signal for *x*	*Task*_*x*_	sin [0.3·*τ* + 1]·50 + 100
task signal for *ϕ*	*Task*_*ϕ*_	cos [0.1·*τ* + 1]·60 + 100
DMP time constant	*α*_*dmp*_	0.75
DMP time constant	*β*_*dmp*_	0.1875
DMP time constant	*α*_*ξ*_	1
Number of DMP kernels	*M*	50
scaling factor	*α*_*ω*_	1
kernel width	*σ*_*dmp*_	0.05
error update rate	*μ*_*ols*_	10^−5^
