# Supplementary material for: The Use of Hebbian Cell Assemblies for Nonlinear Computation
Source: Sci Rep. 2015 Aug 7;5:12866. doi: 10.1038/srep12866 (PMC4650703; doi:10.1038/srep12866)
Supplement: Supplementary Text 1 [file srep12866-s1.pdf]

Supplementary Text 1 for "The Use of Hebbian Cell Assemblies for Nonlinear Computation"

Christian Tetzlaff, Sakyasingha Dasgupta, Tomas Kulvicius, Florentin Wörgötter

Contents

1 Correlation between assembly size and error **2**

2 Setup of motor task **2**

3 Cell assembly formation **3**

3.1 Constraints on cell assembly formation 3
3.2 Controlling assembly outgrowth speed 5
3.3 Cell assembly growth 6
3.4 Contribution of cell assembly halo to computation 10

4 Comparison between adaptive and static neural networks **11**

1 Correlation between assembly size and error

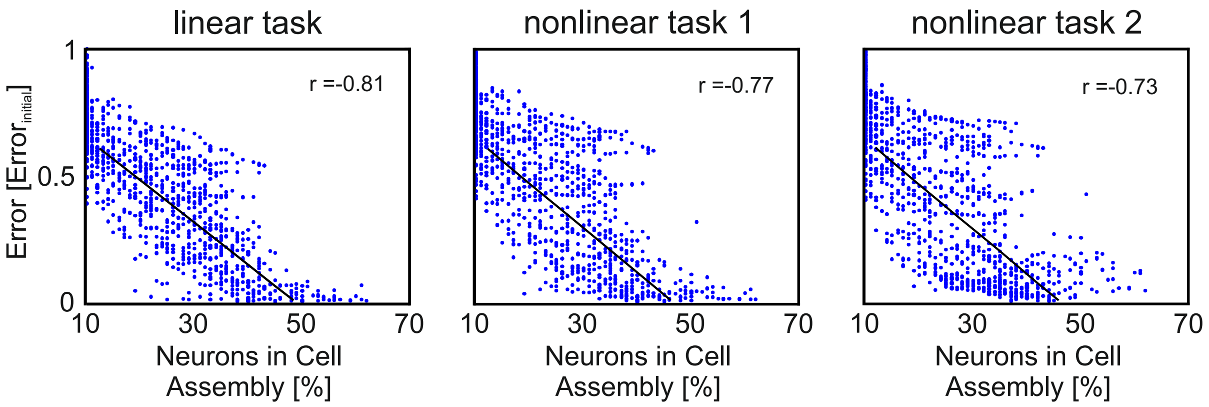

Suppl. Figure 1: Correlation between cell assembly size and computational performance shown by the error. The r-value determines the Pearson correlation coefficient. Solid lines indicate the resulting linear approximation.

2 Setup of motor task

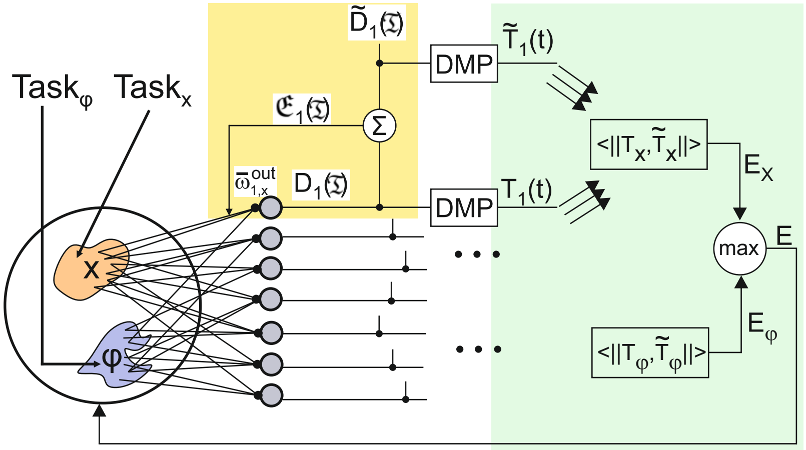

Suppl. Figure 2: Basic setup of the motor task system. Details see Methods section. $< . >_t$ denotes the temporal average.

3 Cell assembly formation

3.1 Constraints on cell assembly formation

The basis of the results shown here is the capability of the network to form competitive cell assemblies. Here we show mathematically that this is a non-trivial process which only happens when certain constraints are fulfilled. Many of the plasticity rules that have been discussed in the literature *cannot easily achieve this*, while the here-used combination of plasticity and scaling seem very well suited for this purpose.

We assume as usual that plasticity has only access to local information such as presynaptic activity F_j, postsynaptic activity F_i and synaptic weight W_{ij} [1, 2]. The self-organized formation of cell assemblies, which compete with each other, implies two major constraints:

(i) synaptic weights inside an assembly have to be larger than outside and

(ii) stimulation of one connection has to induce depression leading to competition at other non-stimulated presynaptic connections.

We can now discuss under which conditions these constraints can be fulfilled.

(i) "Cohesion": A cell assembly is formed by a strong stimulus to a group of units. Thus, the links connecting these stimulated units have to be larger than others (outside the cell assembly). In other words, if two recurrently connected units i and j receive strong inputs, their resulting synaptic weights W_s have to be larger than the ones (w_w) of non- or weakly stimulated units ($W_s > W_w$; Suppl. Fig. 3 A). This constraint implies that the fixed point of the synaptic weight W^*_{ij} has to be:

$$(i) \qquad \boxed{W^*_{ij} \sim F_i \cdot F_j}\,. \tag{1}$$

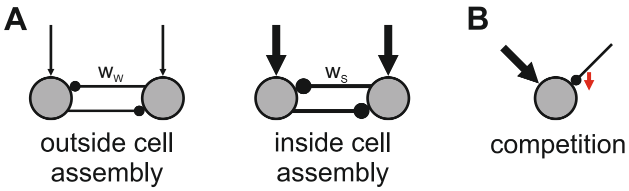

Suppl. Figure 3: Two constraints have to be fulfilled to enable the formation of competitive cell assemblies. (A) The first constraint is that dependent on the input (arrows) the connections outside the cell assembly (left) have to be smaller than inside (right; thickness of lines indicate strength). (B) The second constraint implies that a strong stimulus has to induce a decay of non-stimulated connection, so-called competition.

As Equation 1 looks similar to Hebbian plasticity, it is important to stress that this equation *describes the fixed point* of the weight dynamics and not the dynamics itself and

is therefore not related to the Hebbian rule. Interestingly, all plasticity rules which assure stable weight dynamics by the ratio of pre- and postsynaptic activity,

$$W^*_{ij} \sim \frac{F_j}{F_i} \text{ or } \sim \frac{F_i}{F_j}, \tag{2}$$

as, for instance, the Oja-rule [3], yield $W_s = W_w$ and, therefore, do not allow cell assembly formation as defined above. Furthermore, all plasticity rules controlled by a homeostatic postsynaptic activity mechanism (as, for instance, the BCM-rule [4]), so that the fixed point postsynaptic activity F^*_i has to be equal a constant value Λ as

$$\dot{W}_{ij} = 0 \quad , \text{if } F^*_i = \Lambda, \tag{3}$$

decrease the synaptic weights if units are stimulated. Thus, $W_s < W_w$, and the constraint is not fulfilled.

(ii) "Competition": To assure that cell assemblies do not 'smear' into each other while only one is active, the learning rule has to be competitive. This means that a (strong) stimulation at one connection of postsynaptic unit i induces a decrease of other presynaptic efficacies j (Suppl. Fig. 3 B). Therefore, the fixed point of the non-stimulated connection has to be adapted by the increased postsynaptic activity F_i:

$$(ii) \qquad \boxed{W^*_{ij} \sim \frac{1}{F_i}}. \tag{4}$$

Combining the constraints (i) and (ii): Ultimately the system needs to fullfil both constraints for which we then generically get:

$$(i) + (ii) \qquad \boxed{W^*_{ij} = \left(\frac{\eta F_i \cdot F_j}{\kappa\left(F_i - \Gamma\right)}\right)^{\alpha}} \tag{5}$$

with parameters Γ, κ, η and α. The offset Γ assures that the activity-dependence of competition does not counterbalance the post-synaptic activity-dependence of the cohesion term. η determines the time scale of the cohesion term and κ of the competition term, thus, the ratio $\frac{\eta}{\kappa}$ determines which term dominates the dynamics. The power α determines the gradient of the resulting fixed point-activity function [2]. All learning rules with Equation 5 as fixed point of their weight dynamics are able to form cell assemblies as defined above, for instance, learning rules of following form:

$$\tau_w \dot{W}_{ij} = \eta F_i \cdot F_j + \kappa\ (\Gamma - F_i) \cdot W^{1/\alpha}_{ij}. \tag{6}$$

With $\frac{\tau_w}{\eta} = \tau_H$, $\frac{\tau_w}{\kappa} = \tau_{SS}$, $\Gamma = F^T$, and $\alpha = 1/2$ this equation is equal to the interaction of synaptic plasticity and scaling (Eq. 6 Methods section) used here

$$\frac{dW_{ij}[t]}{dt} = \underbrace{\tfrac{1}{\tau_H} F_i[t] \cdot F_j[t]}_{LTP} + \underbrace{\tfrac{1}{\tau_{SS}} \left(F^T - F_i[t]\right) \cdot \left(W_{ij}[t]\right)^2}_{Synaptic\,Scaling}. \tag{7}$$

3.2 Controlling assembly outgrowth speed

In the previous section we showed under which conditions activity-dependent synaptic adaptation yields cell assembly formation (Eq. 5). Among others, the interaction between synaptic plasticity and scaling fulfills these constraints. The question, however, remains how fast (slow) such processes ought to be to allow for a quick enough acquisition of computational power while still guaranteeing stability.

Thus, apart from the above constraints also the temporal development of such systems is important, captured by the time constant ratio τ_{ratio}, which determines the difference in adaptation speed between synaptic plasticity and scaling. Biological experiments [5] show that synaptic scaling takes about hours to days compared to the time scale of several minutes of synaptic plasticity. Thus, the ratio τ_{ratio} is about 60 (used here) and more. How does this parameter effect the outgrowth of cell assemblies?

Consider a simplified version of the cell assembly outgrowth: two connected units with one unit *ca* as part of the cell assembly and the other *os* outside. The outside unit becomes member of the assembly if the connection from the assembly unit to the outside unit becomes strong. Thus, the growth process of the synaptic efficacy determines the outgrowth speed of the cell assembly. Note, cell assemblies only grow out when they are active ($F_{ca} \approx F_{max}$), otherwise, the activities are so low that synaptic changes can be neglected. Thus,

$$\dot{W}_{os,ca} = \tfrac{1}{\tau_H} F_{os} \cdot F_{max} + \tfrac{1}{\tau_{SS}} \left(F^T - F_{ca}\right) \cdot W_{os,ca}^2. \tag{8}$$

We rewrite this term dependent on the ratio of time scales $\tau_{ratio} = \frac{\tau_{SS}}{\tau_H}$

$$\tau_H \dot{W}_{os,ca} = F_{os} \cdot F_{max} + \tfrac{1}{\tau_{ratio}} \left(F^T - F_{ca}\right) \cdot W_{os,ca}^2. \tag{9}$$

The fixed point of the synaptic weight $W^* = W_{max}$, which has to be reached, depends also on the ratio τ_{ratio}. Thus, we have to estimate the outgrowth relative to the fixed point:

$$\tau_H \dot{W}_{os,ca} = \left(F_{os} \cdot F_{max} + \tfrac{1}{\tau_{ratio}} \left(F^T - F_{ca}\right) \cdot W_{os,ca}^2\right) \cdot W_{max}[\tau_{ratio}] \tag{10}$$

$$= \sqrt{\tau_{ratio} \frac{F_{max}^2}{F_{max} - F^T}} \cdot \left(F_{os} \cdot F_{max} + \tfrac{1}{\tau_{ratio}} \left(F^T - F_{ca}\right) \cdot W_{os,ca}^2\right). \tag{11}$$

During synaptic development the synaptic weight passes two extreme cases: (i) a small initial value ($W_{os,ca} \ll W_{max}$) and (ii) converging to the fixed point ($W_{os,ca} \approx W_{max}$). In each case we can linearize the activity function to $F_{os} \approx c\, F_{max}\, W_{os,ca}$ with constant c_x different for both cases $x \in \{i, ii\}$.

(i): For small weights we can neglect the higher weight-orders in Equation 11.

$$\dot{W}_{os,ca} \approx c_i F_{max}^2 W_{os,ca} \sqrt{\frac{\tau_{ratio}\, F_{max}^2}{F_{max} - F^T}}. \tag{12}$$

Thus, the initial outgrowth depends on the square root of the time scale ratio τ_{ratio}. In other words, if synaptic scaling is too fast compared to plasticity, it dampens the synaptic development and, in turn, the cell assemblies cannot grow out.

(ii): For weights comparable to the maximum weight the higher weight-orders in Equation 11 dominate:

$$\dot{W}_{os,ca} \approx \sqrt{\frac{F_{max}^2}{\tau_{ratio}\left(F_{max} - F^T\right)}} \cdot \left(F^T - c_{ii} F_{max} W_{os,ca}\right) W_{os,ca}^2. \quad (13)$$

So, the weight converges to the maximum depending on $\sqrt{1/\tau_{ratio}}$. Thus, if synaptic scaling is too slow, the system needs long until it will reach its fixed point.

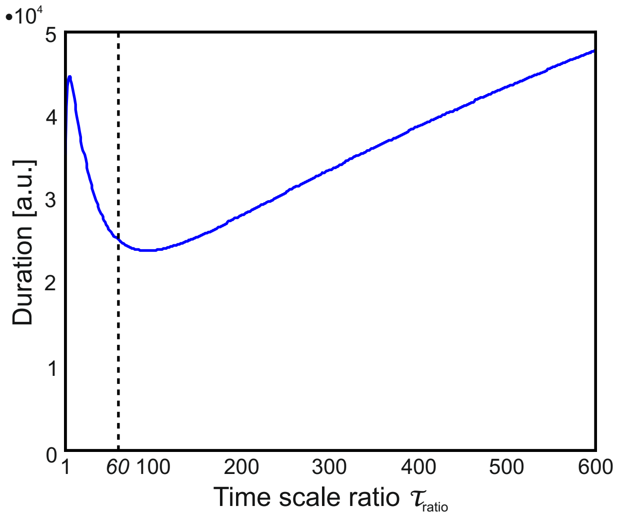

Suppl. Figure 4: The ratio of time scales τ_{ratio} influences the outgrowth speed of cell assemblies. Plotted is the duration the system needs to reach the fixed point (W^*) when the cell assembly is all the time active.

Given case (i) and (ii) the time scale ratio τ_{ratio} should neither be too small (case i) nor too large (case ii). Thus, τ_{ratio} has to be in an intermediate regime. In Suppl. Figure 4 we have measured the time the synaptic weight needs to reach the fixed point (blue) given different values of τ_{ratio}. Clearly their is an intermediate regime of τ_{ratio} (including the here used value) with an optimal outgrowth rate.

3.3 Cell assembly growth

Given an external stimulation (purple in Suppl. Fig. 5 A) to a subset of randomly connected units, the stimulation induces units activities in large parts of the network. As expected, the activity of the directly stimulated units (red) is higher than the activity of

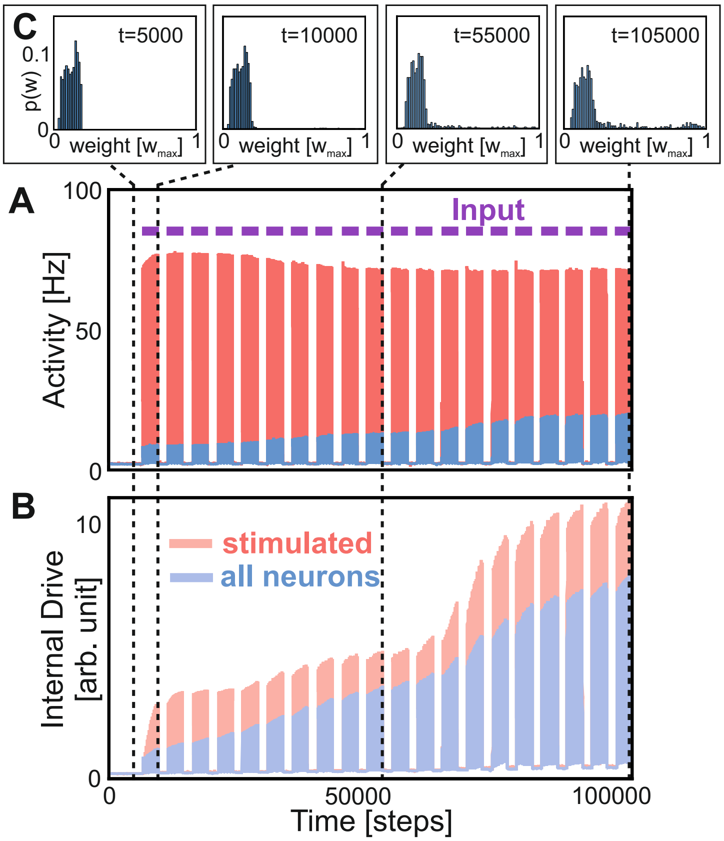

Suppl. Figure 5: Development of units activities, internal drive and weight distribution under sequential learning. (A) A learning signal (purple) induces increased activities in the stimulated units (red). Due to recurrent connections the activity reaches also other parts of the network (blue). With ongoing learning this network activity increases. (B) The increase in activity of non-stimulated units depends on an increase of the internal drive or feedback from the network. (C) The input induces via plasticity changes of the synaptic weights leading to the increase of the internal drive. Thereby, the weight distribution of the network develops from the initial uni-modal ($t = 5000$) to a log-normal one ($t = 105000$).

the non-stimulated ones (blue). However, with ongoing learning the non-stimulated units become more active, too. This is due to an increase of the internal excitatory feedback from the network (Suppl. Fig. 5 B) by increasing synaptic weights (cell assembly outgrowth). Interestingly, the stimulated units receive a stronger feedback from the network than the non-stimulated units. This is due to the specificity of the synaptic changes leading to the formation of cell assemblies. For instance, Suppl. Fig. 6 shows that the first strong connections are formed between the stimulated units (red lines) before connections from the stimulated to non-stimulated (purple) are made and finally also between non-stimulated units (blue). Furthermore, the distribution of the weights develop from an

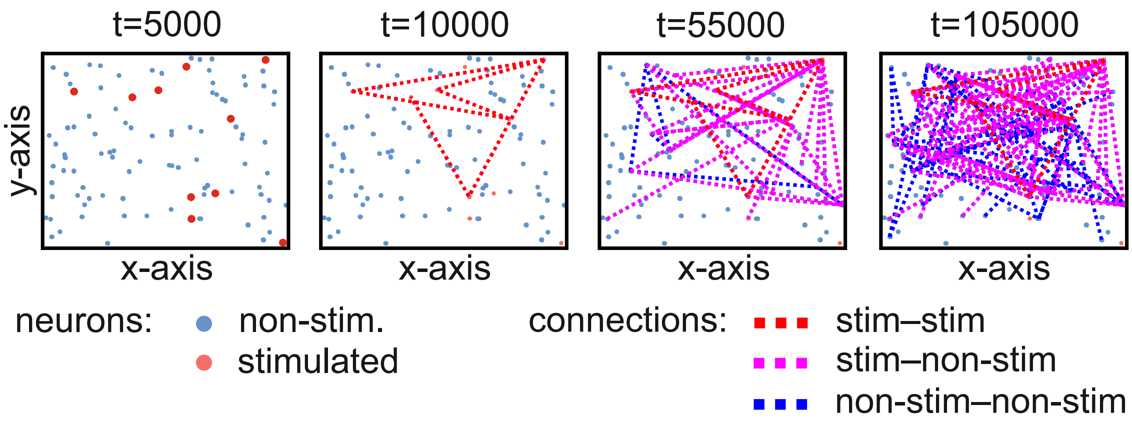

Suppl. Figure 6: Development of a cell assembly. Units are distributed randomly. Lines show connections with weights larger than $W_{max}/2$.

initially uni-modal shape to a log-normal distribution (Suppl. Fig. 5 C) as measured in, e.g., the cortex [6].

The dynamics of the outgrowth of a cell assembly is independent of the exact value of the cell-assembly-threshold θ (Suppl. Fig. 7 A). A detailed analysis of the resulting network also shows that a cluster of strongly interconnected units emerges during learning. For this we calculate the *weighted-shortest path* between each pair of units given the excitatory weight matrix [7]. If two units are strongly connected with each other, the path is short and, therefore, this measure is small. Given the matrix of shortest paths, for visualization, units are now sorted into two groups: those inside the cell assembly (estimated from the threshold-analysis) and the rest. As expected, with ongoing learning, the units, which are part of the cell assembly, also formed a cluster of low-valued *weighted-shortest paths* (red box in Suppl. Fig. 7 B).

Remarkably, the dynamics of the units activities within the cell assembly change with ongoing learning (Suppl. Fig. 7 C). Given a pulsed input to the previously stimulated units, the cell assembly units initially respond in a highly correlated manner. However, the temporal structure of the units activities within a response becomes more complex with growing assembly size. Such complex units activations can then indeed serve as a basis for computation. The assembly responses get also longer and they exceed the input duration, but eventually die down to baseline. This decay indicates that the system is in a subcritical regime [8, 9](comparable to the AI-state [10]) and does not show chaotic [11] or persistent [12] dynamics.

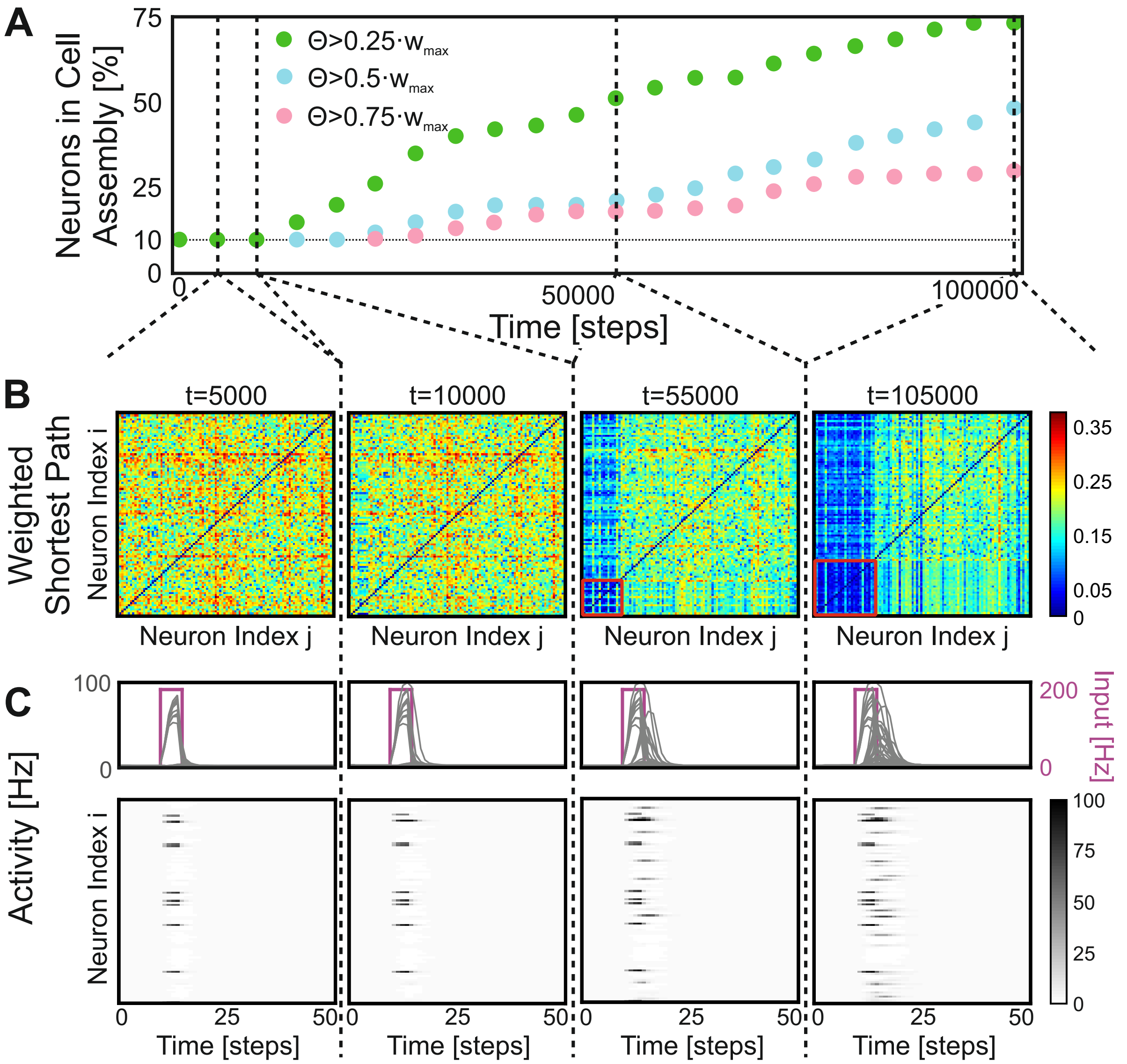

Suppl. Figure 7: Learning induces formation of cell assemblies with diverse units activities. (A) Independent on the θ-threshold the number of units within the cell assembly increases with ongoing learning. (B) Also the shortest paths between units show that a growing cluster emerges (red box). (C) Given a pulse input, the units activities within the cell assembly become more diverse in space and time with increasing size.

3.4 Contribution of cell assembly halo to computation

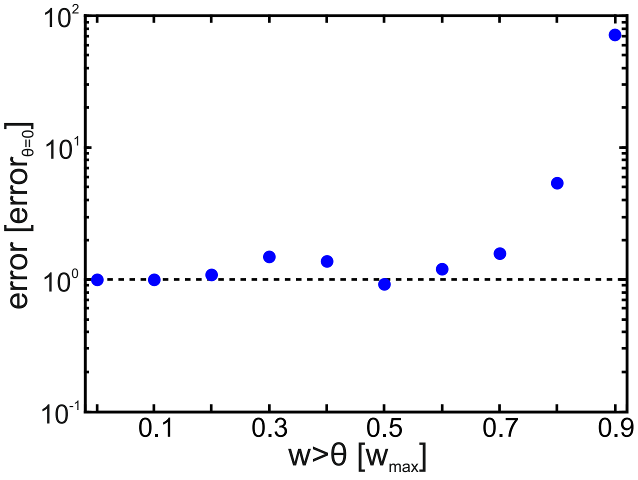

Suppl. Figure 8: Strong connections contribute most to the computational capacity of the cell assembly. By deleting all connections below a threshold θ, we test the contribution of the remaining, strong connections to the nonlinear calculations (here we used nonlinear task 1). The error increases dramatically if connections with weights larger than $0.7 \cdot W_{max}$ are deleted (core of the cell assembly). Please note the logarithmic error-axis.

The outgrowth of the cell assembly clearly supports the performance of the network to compute and solve non-linear tasks. However, it is not clear which part of the cell assembly contributes most to the computation. Therefore, for the final network in Figure 1 main text, we deleted all connections below a threshold θ and tested the computational power of the resulting network. We repeated this procedure for several different thresholds (Suppl. Figure 8). As expected, the strong connections ($W > 0.7 \cdot W_{max}$) contribute most to the computational power of the system as the error increases dramatically by deleting them. Thus, the core of the cell assembly with strongly interconnected units is the most important part. The halo (with smaller synaptic weights) contributes only a little bit to the performance of the system.

4 Comparison between adaptive and static neural networks

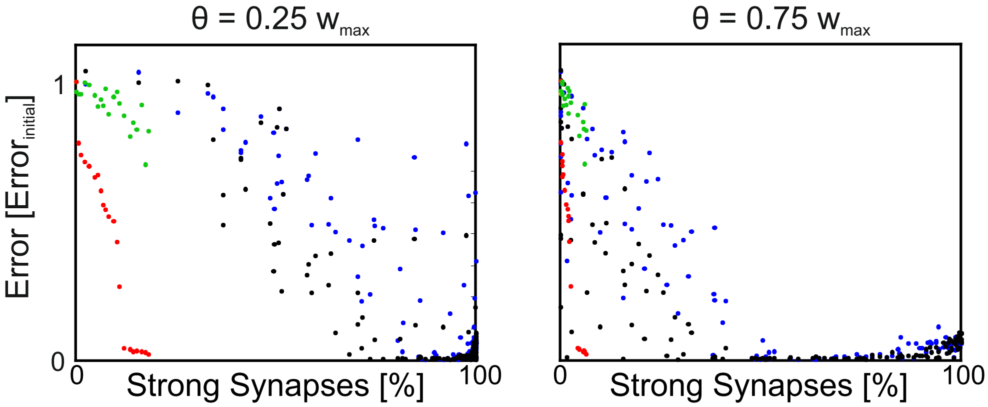

Suppl. Figure 9: Comparison between the adaptive network and random static networks for different weight thresholds θ. Panels are as in main text Figure 2 A with thresholds $\theta = 0.25 \cdot W_{max}$ (left) and $\theta = 0.75 \cdot W_{max}$ (right). Independent of the threshold the number of connections needed in the adaptive network is significantly smaller than for random static ones. Here only one topology is shown.

References

[1] Gerstner, W. and Kistler, W. M. Mathematical formulations of hebbian learning. *Biol. Cybern.* **87**, 404–415 (2002).

[2] Tetzlaff, C., Kolodziejski, C., Timme, M., and Wörgötter, F. Synaptic scaling in combination with many generic plasticity mechanisms stabilizes circuit connectivity. *Front. Comput. Neurosci.* **5**, 47 (2011).

[3] Oja, E. A simplified neuron model as a principal component analyzer. *J. Math. Biol.* **15**, 267–273 (1982).

[4] Bienenstock, E. L., Cooper, L. N., and Munro, P. W. Theory for the development of neuron selectivity: orientation specificity and binocular interaction in visual cortex. *J. Neurosci.* **2**, 32–48 (1982).

[5] Turrigiano, G. G., Leslie, K. R., Desai, N. S., Rutherford, L. C., and Nelson, S. B. Activity-dependent scaling of quantal amplitude in neocortical neurons. *Nature* **391**, 892–896 (1998).

[6] Song, S., Sjöström, P. J., Reigl, M., Nelson, S. B., and Chklovskii, D. B. Highly nonrandom features of synaptic conectivity in local cortical circuits. *PLoS Biol.* **3(3)**, e68 (2005).

[7] Rubinov, M. and Sporns, O. Complex network measures of brain connectivity: uses and interpretations. *Neuroimage* **52**, 1059–1069 (2010).

[8] Tetzlaff, C., Okujeni, S., Egert, U., Wörgötter, F., and Butz, M. Self-organized criticality in developing neuronal networks. *PLoS Comput. Biol.* **6(12)**, e1001013 (2010).

[9] Priesemann, V. et al. Spike avalanches in vivo suggest a driven, slightly subcritical brain state. *Front. Syst. Neurosci.* **8**, 108 (2014).

[10] Brunel, N. Dynamics of networks of randomly connected excitatory and inhibitory spiking neurons. *J. Physiol.* **94**, 445–463 (2000).

[11] Sussillo, D. and Abbott, L. F. Generating coherent patterns of activity from chaotic neural networks. *Neuron* **63**, 544–557 (2009).

[12] Barak, O. and Tsodyks, M. Working models of working memory. *Curr. Opin. Neurobiol.* **25**, 20–24 (2014).
